# Latent trait modeling of tau neuropathology in progressive supranuclear palsy

**DOI:** 10.1007/s00401-021-02289-0

**Published:** 2021-02-26

**Authors:** Naomi Kouri, Melissa E. Murray, Joseph S. Reddy, Daniel J. Serie, Alexandra Soto-Beasley, Mariet Allen, Minerva M. Carrasquillo, Xue Wang, Monica Casey Castanedes, Matthew C. Baker, Rosa Rademakers, Ryan J. Uitti, Neill R. Graff-Radford, Zbigniew K. Wszolek, Gerard D. Schellenberg, Julia E. Crook, Nilüfer Ertekin-Taner, Owen A. Ross, Dennis W. Dickson

**Affiliations:** 1 Department of Neuroscience, Mayo Clinic, 4500 San Pablo Road, Jacksonville, FL 32224 USA; 2 Department of Health Sciences Research, Mayo Clinic, Jacksonville, FL USA; 3 VIB-UAntwerp Center for Molecular Neurology, Antwerp, Belgium; 4 Department of Neurology, Mayo Clinic, Jacksonville, FL USA; 5 Department of Pathology and Laboratory Medicine, University of Pennsylvania, Philadelphia, PA USA

**Keywords:** Progressive supranuclear palsy, *MAPT*, *MOBP*, Tau, Latent traits

## Abstract

Progressive supranuclear palsy (PSP) is the second most common neurodegenerative Parkinsonian disorder after Parkinson’s disease, and is characterized as a primary tauopathy. Leveraging the considerable clinical and neuropathologic heterogeneity associated with PSP, we measured tau neuropathology as quantitative traits to perform a genome-wide association study (GWAS) within PSP to identify genes and biological pathways that underlie the PSP disease process. In 882 PSP cases, semi-quantitative scores for phosphorylated tau-immunoreactive coiled bodies (CBs), neurofibrillary tangles (NFTs), tufted astrocytes (TAs), and tau threads were documented from 18 brain regions, and converted to latent trait (LT) variables using the R *ltm* package. LT analysis utilizes a multivariate regression model that links categorical responses to unobserved covariates allowing for a reduction of dimensionality, generating a single, continuous variable to account for the multiple lesions and brain regions assessed. We first tested for association with PSP LTs and the top PSP GWAS susceptibility loci. Significant SNP/LT associations were identified at rs242557 (*MAPT* H1c sub-haplotype) with hindbrain CBs and rs1768208 (*MOBP*) with forebrain tau threads. Digital microscopy was employed to quantify phosphorylated tau burden in midbrain tectum and red nucleus in 795 PSP cases and tau burdens were used as quantitative phenotypes in GWAS. Top associations were identified at rs1768208 with midbrain tectum and red nucleus tau burden. Additionally, we performed a PSP LT GWAS on an initial cohort, a follow-up SNP panel (37 SNPs, *P* < 10^–5^) in an extended cohort, and a combined analysis. Top SNP/LT associations were identified at SNPs in or near *SPTBN5*/*EHD4*, *SEC13*/*ATP2B2*, *EPHB1*/*PPP2R3A*, *TBC1D8*, *IFNGR1*/*OLIG3*, *ST6GAL1*, *HK1*, *CALB1*, and *SGCZ*. Finally, testing for SNP/transcript associations using whole transcriptome and whole genome data identified significant expression quantitative trait loci at rs3088159/*SPTBN5*/*EHD4* and rs154239/*GHRL*. Modeling tau neuropathology heterogeneity using LTs as quantitative phenotypes in a GWAS may provide substantial insight into biological pathways involved in PSP by affecting regional tau burden.

**Supplementary Information:**

The online version contains supplementary material available at 10.1007/s00401-021-02289-0.

## Introduction

Progressive supranuclear palsy (PSP) is an atypical Parkinsonian disorder where patients typically exhibit early unexplained falls, vertical gaze palsy, axial rigidity, and levodopa unresponsive parkinsonism. PSP is a primary tauopathy because on neuropathologic examination there is predominately hyperphosphorylated, aggregated tau protein. Microtubule associated protein tau encoded by the *MAPT* gene binds to microtubules and is important for maintaining neuronal morphology and function.

PSP neuropathologic features include tau-immunoreactive neuronal and glial lesions in the basal ganglia, diencephalon, and brainstem with variable involvement of the neocortex. Macroscopic examination in PSP reveals pigment loss in the substantia nigra and atrophy of multiple brain regions including superior cerebellar peduncle, subthalamic nucleus, hilus of the cerebellar dentate, and midbrain with dilation of the aqueduct of Sylvius. Neuropathologic diagnostic criteria for PSP require the presence of tau neurofibrillary tangles in the most affected nuclei which are the globus pallidus, subthalamic nucleus, and substantia nigra [19, 31]. Tufted astrocytes (TA) are astrocytic tau lesion found consistently in the motor cortex and striatum in PSP [34, 38] and oligodendroglial coiled bodies (CB), often accompanied by neuropil threads in the white matter of the diencephalon, brainstem, and cerebellum [5].

Aside from the typical PSP profile, there exists neuropathologic variants of PSP [14]. Reflecting the heterogeneity of PSP tau pathology distribution, PSP patients can present with a variety of clinical presentation including frontotemporal dementia, spastic paraparesis, progressive apraxia if speech, corticobasal syndrome, primary gait failure with freezing, and a Parkinsonian-like syndrome that is initially responsive to levodopa therapy [1, 51]. In patients with primary gait failure with freezing there is greater tau pathology in the globus pallidus, diencephalon, and brainstem and more specifically, pallido-nigro-luysial atrophy with very mild if any cortical pathology. PSP presenting with typical Parkinson’s disease features may have milder tau pathology overall.

Although there are rare familial PSP cases [16] and an estimated 10–15% positive family history of neurological disorder, PSP is considered a sporadic disorder. Despite the majority of PSP patients reported to have negative family history, there is a strong genetic risk factor located on chromosome 17q21 composed of a ~ 1.2 Mb inversion surrounding *MAPT* termed the H1 and H2 haplotypes [6, 12]. The *MAPT* locus associated with PSP risk was further analyzed and identified subhaplotypes in the H1 haplotype background that associate with risk of developing disease [40, 43]. However, the H1 haplotype frequency is ~ 80% in healthy control populations of European ancestry [15] and therefore alone, H1 is not sufficient to cause disease. The H1 and H1c subhaplotype association with PSP was confirmed and further strengthened in the largest PSP genetic association study to date [20]. Additionally, five novel PSP susceptibility loci have been identified at *MOBP*, *STX6*, *EIAF2AK3*, *SLCO1A2I,* and *DUSP10* [11, 46]. These new susceptibility loci identified in the PSP GWASs have the potential to reveal new insights into PSP, but need to be further studied to understand their role in disease pathogenesis.

Latent variable models are based on Item Response Theory (IRT) and are commonly used to link latent traits (LT) to unobserved covariates using dichotomous or polytomous manifest variables [45]. This allows one to draw conclusions from complex datasets and summarize the information by a reduction of dimensionality, often applied to psychometric testing and tests that measure personality traits, cognitive traits, moods, and behavioral dispositions. Semejima’s graded response model (GRM) is an extension of the basic IRT model, which analyzes polytomous rather than dichotomous responses. Here we apply the GRM to semi-quantitative tau neuropathology (i.e. polytomous variables) to generate a LT variable summarizing the tau neuropathology profile for each individual PSP case using the *ltm* R package [44]. This approach to reducing the complex dataset of four tau lesions in 18 brain regions per PSP case allows for quantitative trait testing in genetic association studies.

## Methods

### Neuropathologic evaluation

Neuropathologically-confirmed PSP cases from the Mayo Clinic Florida brain bank were evaluated by one neuropathologist (Dennis W. Dickson) and selected for inclusion in this study based on the completeness of tau neuropathology data and availability of frozen tissue for DNA extraction. Sixteen sections from one formalin-fixed hemisphere were systematically sampled, paraffin embedded, and cut into 5 μm slices. Tissue sections were deparaffinized then pretreated with steam in deionized water for 30 min and stained with anti-phosphorylated tau CP13 (1:1000, pSer202, kind gift from Dr. Peter Davies, Feinstein Institutes for Medical Research), or pretreated with steam in tris pH = 9 buffer for 30 min and stained with anti-MOBP (1:250, Thermo Fisher Scientific cat# PA5-72493) using a Lab Vision Autostainer 480S (Thermo Fisher Scientific, Waltham, MA, USA). Immunostained slides were subsequently counterstained with hematoxylin. All postmortem samples were acquired with appropriate ethical approval and this study was approved by the Mayo Clinic Institutional Review Board.

Semiquantitative tau pathology measures (none = 0, mild = 1, moderate = 2, severe = 3) were assessed from phosphorylated tau immunostained sections in 18 different anatomical structures for the following lesions: NFTs, CBs, TAs, and threads. Brain regions were selected based on those affected in PSP, which include: basal nucleus, caudate putamen, globus pallidus, hypothalamus, motor cortex, subthalamic nucleus, thalamic fasciculus, ventral thalamus, cerebellar white matter, dentate nucleus, inferior olive, locus ceruleus, medullary tegmentum, midbrain tectum, oculomotor complex, pontine base, pontine tegmentum, red nucleus, and substantia nigra. Temporal cortex tau pathology scores were also collected but excluded from further analyses because the distribution of scores for the sample set was skewed and incomplete due to this region being minimally affected in PSP.

### Latent variable modeling

Polytomous ordinal data was handled using the Graded Response Model (GRM) within the *ltm* R package [44]. Semiquantitative tau pathology data was available for 906 patients and were used to generate LTs although some were later excluded from analyses based on genome-wide genotyping quality control. The 72 total pathology measures (18 brain regions and 4 tau lesions per region) were each on a 0 to 3 scale and were used to create an overall score for degree of pathology based on a latent trait approach. Temporal cortex exhibited highly skewed or bimodal distributions and were therefore excluded from LT calculations. The overall latent variable score is an estimate of an assumed underlying level of pathology severity that all individual scores are dependent on or correlated with. Brain regions were divided into “forebrain” and “hindbrain” categories defined by the mesencephalic/diencephalic junction. Twelve LT measures were used as intermediate phenotypes and these included CB overall, CB hindbrain, CB forebrain, NFT overall, NFT hindbrain, NFT forebrain, TA overall, TA hindbrain, TA forebrain, tau threads overall, tau threads hindbrain, tau threads forebrain, Three additional LT measures were generated for overall, forebrain, and hindbrain LTs, which included all four lesions for all brain regions and regions above and below the mesencephalic/diencephalic junction. Information curves are a feature generated as part of the *ltm* package and illustrate the information provided by each brain region as the area under the curve used to calculate LTs.

### Digital neuropathology

IHC slides were scanned at 20X on an Aperio AT2 digital whole slide scanner (Leica Biosystems, Buffalo Grove, IL, USA), which converts slides into high-resolution digital images. Regions of interest were selected based on the overall LT information curve in order to minimize total number of regions analyzed. Midbrain tectum and red nucleus were analyzed at the level of the 3^rd^ cranial nerve on midbrain sections. ImageScope v12.4.2 (Leica Biosystems) software was used to trace regions of interest as annotation layers. A custom color deconvolution macro (v9) was used to analyze CP13 immunoreactivity by detecting only DAB + color profile as a percent strong threshold while calculating all other colors as negative (percent medium and weak thresholds) (Online Resource Fig. 2). Regions of interest on MOBP-immunostained slides from superior frontal cortex and pons were annotated similarly and included corpus callosum, anterior cingulate white matter, superior frontal white matter, deep frontal white matter, and pontine base. A custom IHC nuclear macro (v1) was created to quantify small MOBP-immunoreactive granules in white matter of representative forebrain and hindbrain regions. The macro is able to discriminate nuclei from MOBP granules based on color and size and expressed as a percent 3 + nuclei in the macro (Online Resource File of macro parameters).

### Genotyping

Genome-wide genotyping data were generated using Illumina 660 W-Quad chips and raw intensity files were subjected to cluster analysis in GenomeStudio software (Illumina, San Diego, CA, USA). Quality control of genotyping data was performed at the individual level and then at the SNP level. For quality control, 10 individuals were genotyped in duplicate. Exclusion criteria for individual samples included high genotype failure rate (> 2% excluded 4 samples) and cryptic relatedness or sample duplicates (all samples met these criteria). Gender inconsistencies were assessed by chromosome X genotypes which excluded 29 individuals based on observed and expected gender. Exclusion criteria for markers included minor allele frequency (7420 SNPs were removed because of minor allele frequency < 0.1%) and high genotype failure rates (33,191 SNPs were removed because of genotype failure rates > 2%).

Selection criteria for the extended PSP cohort follow-up SNPs (Online Resource Table 1) include LT/genotype associations with *P* < 10^–5^ (*N* = 37) and that the SNPs were in Hardy–Weinberg equilibrium (*P* < 0.05). Based on these top loci, an additional ~ 550 SNPs for each LT (*P* < 0.001) were further scrutinized by determining the linkage disequilibrium structure between the SNP clusters. Based on this, the most significant SNPs were included and those in tight linkage disequilibrium (*R*^2^ > 0.95) with these top SNPs were excluded from further analysis. Genotyping of Stage 2 SNPs was performed on the MassArray iPlex platform (Agena Bioscience, San Diego, CA, USA). Genetic variants that were not compatible with iPlex technology were analyzed by individual pre- and custom-designed Taqman assays following manufacturer protocol (Thermo Fisher Scientific, Waltham, MA, USA) or by direct sequencing analyses. Sanger sequencing was performed on PCR reactions. PCR products were purified using AMPure (Beckman Coulter, Brea, CA, USA) then sequenced in both directions using the BigDye Terminator cycle sequencing kit (Thermo Fisher Scientific, Waltham, MA, USA). Sequencing reactions were purified using CleanSEQ (Beckman Coulter, Brea, CA, USA) and analyzed on an ABI3730xl Genetic Analyzer. AMPure and CleanSEQ purifying reactions were performed on a Biomek FX Laboratory Automation Workstation (Beckman Coulter, Brea, CA, USA). Base calling, sequence alignments and heterozygote detection will be performed using Sequencher v4.8 (Gene Codes, Ann Arbor, MI, USA).

### Relatedness and population stratification

Relationships among samples were evaluated using KING-robust [33] in PLINK2 [10] (v2.00a3LM) using the “*–*make-king-table*”* flag. Samples related up to the 3rd degree (Kinship coefficient ≥ 0.0442) were identified and one sample from each pair of relateds with the best call rate was retained. After resolving relatedness, the underlying population substructure was evaluated using Eigenstrat [39, 41] to identify and remove population outliers. Eigenstrat was set to remove outliers of up to 6 standard deviations of the top 10 principal components (PCs) over five iterations, while refitting PCs after each iteration of outlier removal. While one sample was removed for relatedness and six samples identified as population outliers were also removed.

### Imputation

Samples and variants that passed QC were imputed to the Haplotype Reference Consortium (HRC) reference panel [35] using Minimac4 imputation algorithm [13] with Eagle phasing [32] implemented by the Michigan Imputation Server (MIS, v1.5.7) [13]. Prior to imputation, the strand and position of variants were harmonized with the HRC reference panel (r1.1 2016) using tools provided by the McCarthy Group at the University of Oxford, UK (https://www.well.ox.ac.uk/~wrayner/tools/index.html). Harmonized genotypes were exported to VCF files and uploaded to the MIS for imputation. Given that the imputation process replaces original genotypes with imputed ones, an in-house script was utilized to reinstate the original genotypes to the VCF after imputation. Variant dosages from imputation were imported into PLINK 2 (v2.00a3LM) using the “dosage = DS” flag. Variants with in imputation *R*^2^ ≥ 0.9 and a minor allele frequency (MAF) ≥ 2% were retained for LT GWAS.

### Mayo RNAseq and WGS datasets

The Mayo RNAseq dataset comprises transcriptome measures from temporal cortex (TCX) and cerebellum (CER); RNA isolation, data collection, sequencing alignment, counting and QC has been described in detail elsewhere [2, 4]. Gene counts were normalized using conditional quantile normalization (CQN) [18]. DNA **s**ample processing and sequencing protocols are described on the AMP-AD knowledge portal: https://adknowledgeportal.synapse.org/, Synapse ID: syn10901601. FastQ files from sequencing were processed through Mayo Clinic’s GenomeGPS pipeline. Briefly, reads were aligned to the reference (hg19) using Novoalign and variant calling and genotyping was performed while implementing GATK’s Best Practices Workflow [48]. Samples were QC-ed for coverage (at least 90% covered at 10 × and 50% covered at 30x), genotyping quality (median GQ of 99), call rate (95%), transition to transversion (Ti/Tv) ratio (between 2 and 2.1), sex (PLINK inbreeding coefficient of the X-chromosome for males > 0.7 and females < 0.3) and contamination (VerifyBamID [25] FREEMIX score less than 0.02). Subsequently, samples were evaluated for relatedness up to 3^rd^ degree, population substructure and sequencing batch effects. Variants passing VQSR filter, having a genotyping rate of 95% or more, a Bonferroni adjusted Hardy–Weinberg *p* value greater than 0.05 in controls and BLAT [26] score less than four were retained for downstream analysis. All 349 samples and 19,357,792 variants passed QC. Genotypes were extracted from VCF files using PLINK [10]. CQN gene expression values and accompanying WGS genotypes of variants having a minor allele frequency equal to or greater than 5% in samples with expression data were subsequently utilized for eQTL analysis.

### Statistical analysis

Variants within ± 1 Mb of the Ensembl gene locus were tested for association with CQN gene expression levels (eQTL) using a linear mixed model implemented with the *lme4* package [7] in R statistical software version 3.5.2. CQN expression value was the dependent variable; variant dosage (0, 1 or 2) was the independent variable. All QTL models were adjusted for diagnosis, sex, age at death, RIN, *APOE* ε4 dosage, tissue source, flowcell and the first three principle components, with flowcell being the random effects variable. Denominator degrees of freedom for test statistic was obtained using Kenward-Roger [27] restricted maximum likelihood approximation in the *lmerTest* package [30] in R. False-discovery rate adjusted (Benjamini-Hochberg) q-values were calculated in R for all tested eQTLs.

For PSP LT GWAS study the initial PSP cohort (*n* = 498) with genome-wide genotyping data was tested for genetic association with LTs by linear regression under an additive model using age at death and sex as covariates with PLINK [42]. With *α* = 0.05 and Bonferroni correction for the 559,348 SNPs tested in stage 1, a *P* value of 9.0 × 10^–8^ is required for 'genome-wide' significance. For the combined analysis, an extended PSP cohort was tested for association with PSP LTs by the same method as the primary PSP cohort, and followed by a combined analysis using PLINK. The PSP LT GWAS was re-analyzed using imputed genotypes in the initial PSP cohort (*n* = 472). Variant dosages were tested for their association with latent traits in PLINK2 (v2.00a3LM) using –glm flag using age at death, sex, and first four principle components to adjust for population substructure. Results were annotated using Annovar [49].

## Results

### Latent trait association with top PSP susceptibility loci

**Fig. 1:**
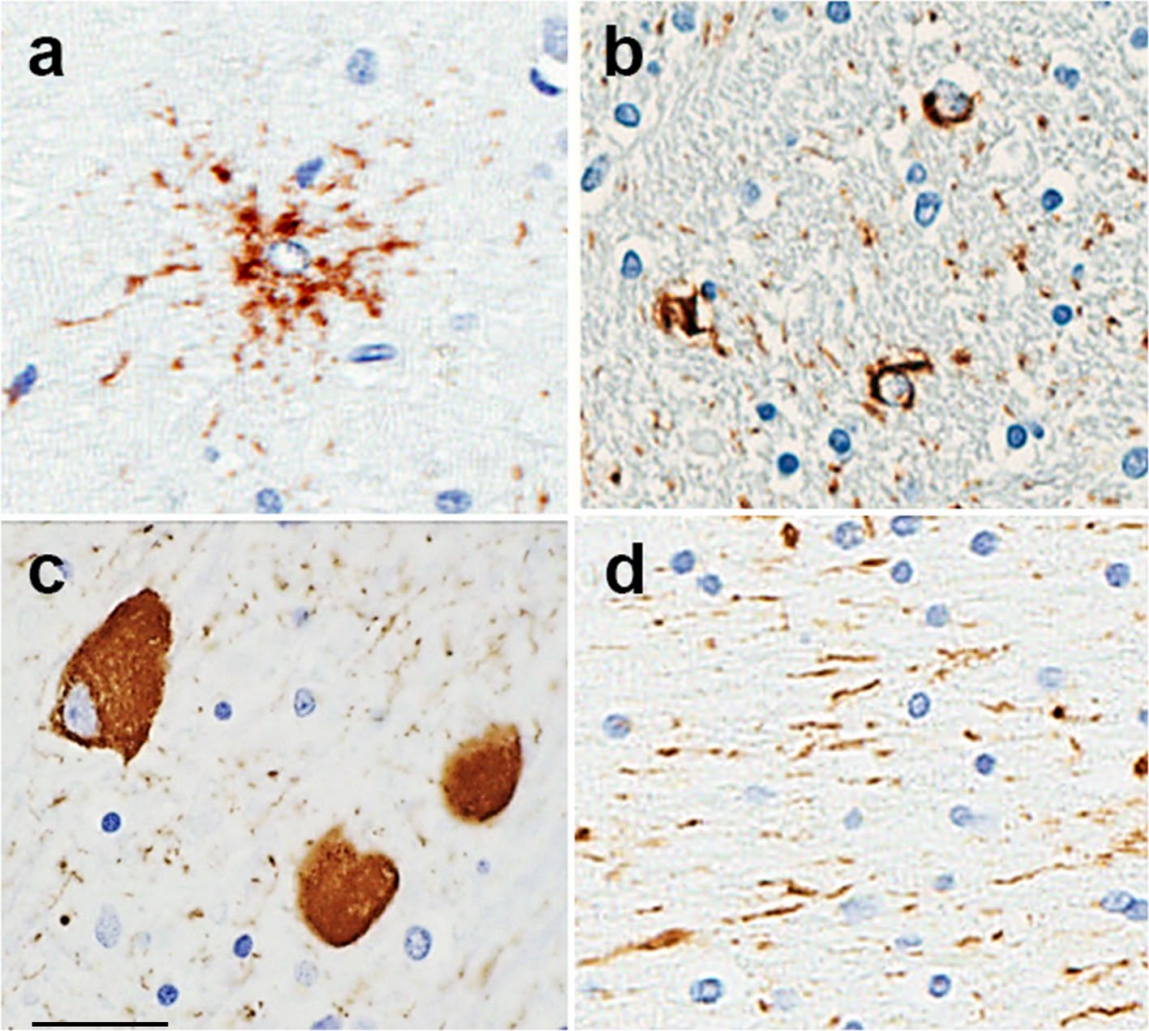
Tau-immunoreactive lesions in PSP. Characteristic phosphorylated tau-immunoreactive lesions in PSP were used to generate latent trait scores from semi-quantitative scores using the *ltm* R package. Tufted astrocytes (**a**), oligodendroglial coiled bodies (**b**), neurofibrillary tangles (**c**), and tau neuropil threads (**d**). Scale bar = 30 μm

**Fig. 2:**
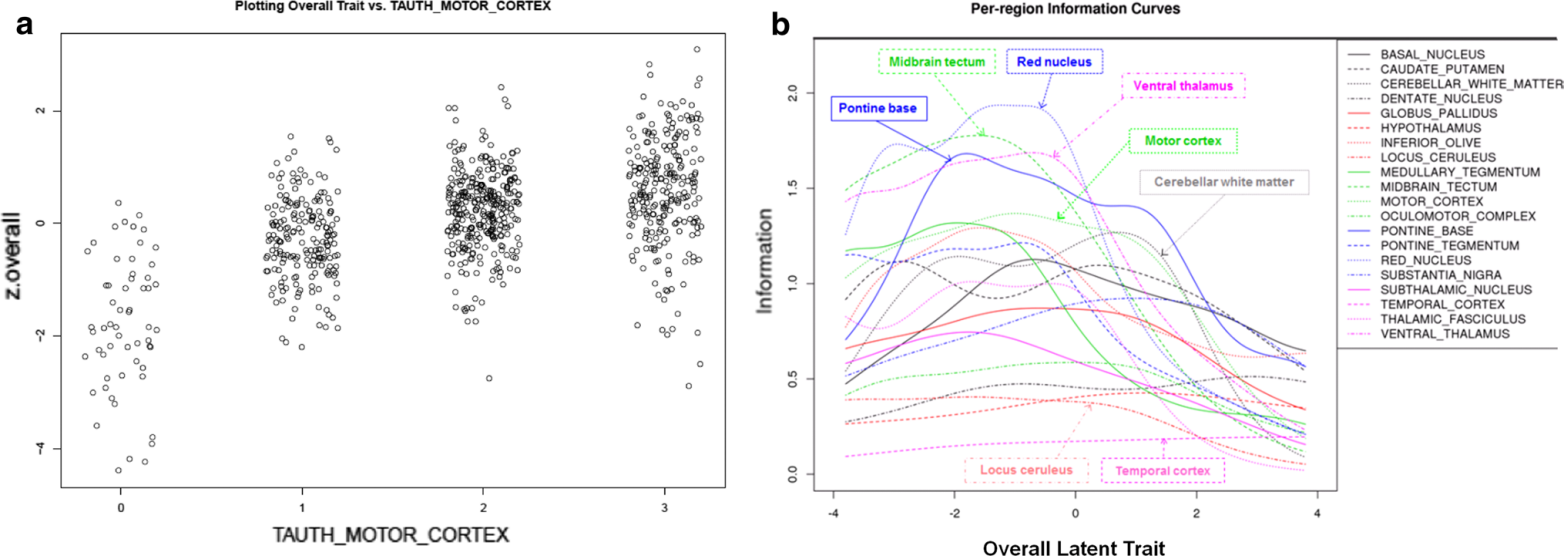
Latent variable modeling of PSP neuropathology. This representative scatter plot shows the relationship of latent traits to semi-quantitative tau pathology scores. Information from the original data is not lost as there is an increase in the overall LT score as semi-quantitative score increase for tau threads in the motor cortex (**a**). The ltm R package generates Information plots in order to understand the contribution of information provided to the LT score per item (i.e. anatomical structures). The Overall Item Information Curve indicates that the items locus ceruleus and temporal cortex provide little information in the whole latent trait continuum, whereas midbrain structures and motor cortex provide the greatest amount of information (**b**)

Semiquantitative tau pathology measures (none = 0, mild = 1, moderate = 2, severe = 3) were assessed from CP13 immunostained sections in 18 different anatomical structures for the following lesions: tufted astrocytes (TA) (Fig. 1a) oligodendroglial coiled bodies (CB) (Fig. 1b), neurofibrillary tangles (NFT) (Fig. 1c), and tau neuropil threads (Fig. 1d). PSP LTs were then generated from these semi-quantitative tau pathology scores for the study cohort (*N* = 904, Table 1) using the R *ltm* package. LTs were generated for each tau lesion type, separated into hindbrain and forebrain regions, and an overall LT variable *i.e.* all brain regions per lesion. Plotting semi-quantitative scores against LTs, shows that the original information is not lost, as there is an increase in LT as semi-quantitative score increase (Fig. 2a). This resulted in a total of 16 LT phenotypes tested for genetic association. As part of the *ltm* R package, Information plots are generated with the LT scores in order to display the contribution of information provided to the LT score per item (i.e. anatomical structures). The Overall Item Information Curve indicates that the items locus ceruleus and temporal cortex provide little information in the whole latent trait continuum (Fig. 2b). The greatest information is provided by the items midbrain tectum, red nucleus, pontine base, ventral thalamus, and motor cortex which is consistent with what we observe in PSP tau neuropathology. For example, tau pathology burden in locus ceruleus and temporal cortex are not highly variable, whereas the midbrain structures and motor cortex have considerable heterogeneity in affection, hence the hindbrain- and forebrain-predominant PSP subtypes. Information Curve plots for NFTs, CBs, TAs, and Threads show variability in item information per lesion type (Online Resource Fig. 1).

**Table 1:** Demographics of PSP cases

PSP Cohort	Samples analyzed	M: F	Female %	Age at death (y)		
				Mean age	Range	s.d
Initial	481	245: 235	49	75.0	46–98	8.0
Extended	401	233: 168	43	74.7	45–91	7.5
Combined	882	478: 403	46	74.9	45–98	7.8

*M*, male; *F*, female; *s.d.*, standard deviation

**Fig. 3:**
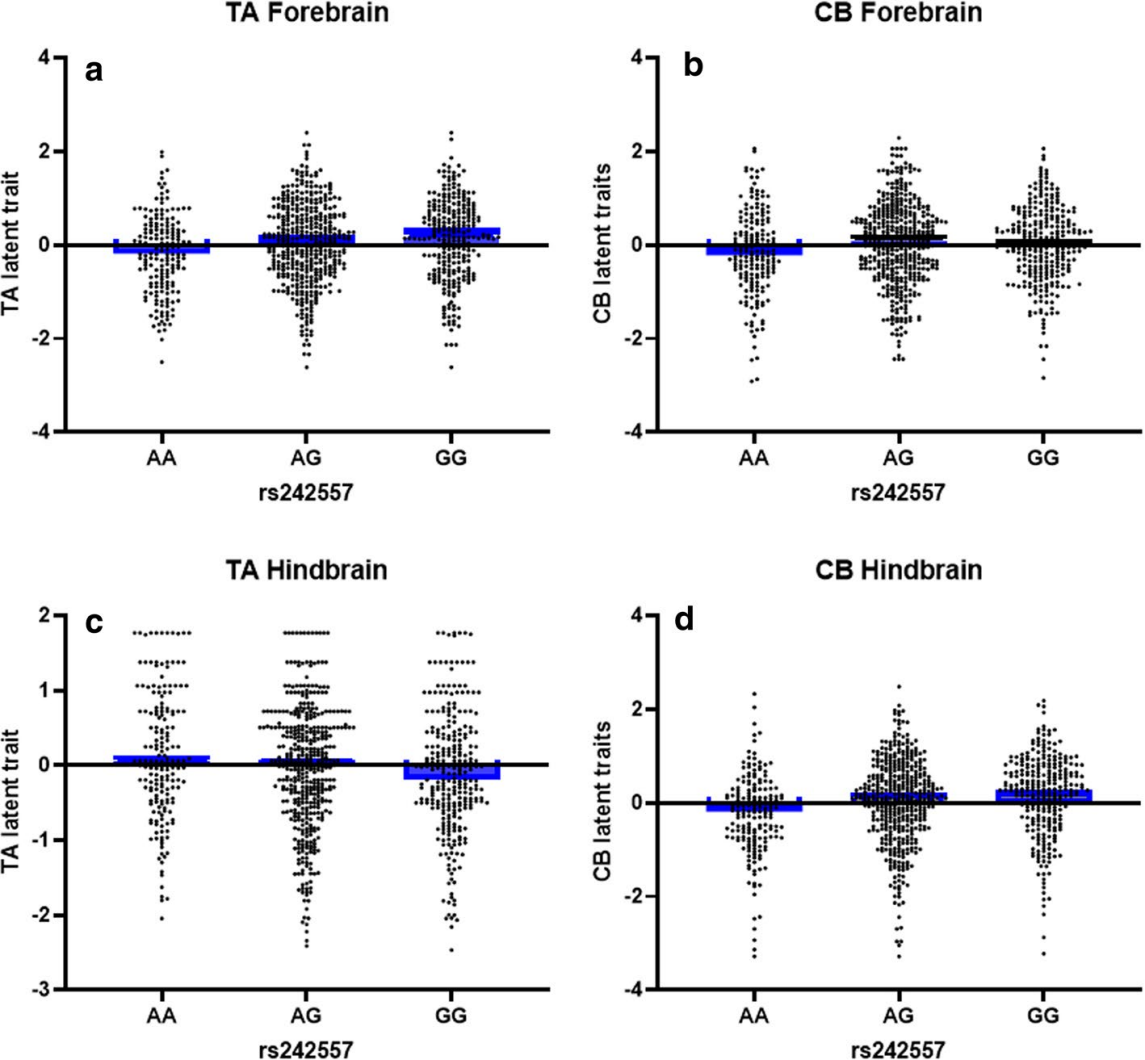
Latent trait scores stratified by rs242557 genotypes indicate an association with glial tau burden. The rs242557 *MAPT* H1c haplotype-tagging SNP is associated with variability in CB and TA burden. LT measures were generated from semi-quantitative scores of tau-immunoreactive CBs and TAs in a large PSP cohort and plotted stratified by rs242557 genotype. These LT measures were used as phenotypes in linear regression analysis. The “A” risk allele is associated with decreased TA burden in the forebrain (**a**), increased TA burden in the hindbrain (**b**), and decreased forebrain (**c**) and hindbrain (**d**) CB burden. *LT*, latent trait; *CB*, coiled bodies; *TA*, tufted astrocytes; Blue bars represent the median

PSP LTs were then used as quantitative traits to test for genetic association using linear regression under an additive model using age and sex as covariates. We tested for association with the top PSP susceptibility loci at *MAPT* (rs8070723 for the H1H2 haplotype; rs242557 for H1c haplotype), *MOBP* (rs1768208), *EIF2AK3* (rs7571971), and *STX6* (rs1411478), *SLCO1A2* (rs11568563), and *DUSP10* (rs6687758) [11, 20, 46]. Meta-analysis of these five SNPs resulted in significant associations with rs242557 and LTs measuring glial tau lesion load (Table 2). The regional effect of genotype on tau pathology burden in PSP can be visualized upon stratification by rs242557 genotype where “A” is the risk allele and “G” the protective allele (Fig. 3). The H1c haplotype appears to have regional effects on tau burden in PSP. Each copy of the “A” risk allele is associated with less TA burden in forebrain structures (*P* = 1.56 × 10^–4^, Beta = − 0.159) (Fig. 3a), yet greater TA burden in hindbrain structures (*P* = 2.18 × 10^–3^, Beta = 0.121) (Fig. 3b). Whereas the rs242557 “A” allele is associated with decreased CB load in both forebrain (*P* = 8.98 × 10^–2^, Beta = − 0.066) (Fig. 3c) and hindbrain (*P* = 1.03 × 10^–6^, Beta = − 0.201) (Fig. 3d).

**Table 2:** Latent trait association result with *MAPT* H1c haplotype-tagging SNP rs242557 and *MOBP* SNP rs1768208 in a combined analysis of the initial and extended PSP cohorts

rs242557						
Latent trait	Initial		Extended		Combined	
	BETA	*P* value	BETA	*P* value	BETA	*P* value
CB hind	− 0.196	3.06 × 10^–4^	− 0.207	1.20 × 10^–3^	− 0.201	1.03 × 10^–6^
CB overall	− 0.163	2.31 × 10^–3^	− 0.154	2.44 × 10^–2^	− 0.160	1.42 × 10^–4^
TA fore	− 0.222	6.43 × 10^–5^	− 0.071	0.28	− 0.159	1.56 × 10^–4^
TA overall	− 0.208	2.92 × 10^–4^	− 0.089	0.20	− 0.160	2.82 × 10^–4^
TA hind	0.145	4.77 × 10^–3^	0.086	0.17	0.121	2.18 × 10^–3^
Hind overall	− 0.120	3.16 × 10^–2^	− 0.117	7.97 × 10^–2^	− 0.119	5.42 × 10^–3^

rs1768208						
Latent trait	Initial		Extended		Combined	
	BETA	*P* value	BETA	*P* value	BETA	*P* value
Threads fore	0.178	9.68 × 10^–4^	0.157	8.00 × 10^–3^	0.169	2.14 × 10^–5^
Threads overall	0.176	1.44 × 10^–3^	0.104	9.92 × 10^–2^	0.145	4.65 × 10^–4^
Fore overall	0.148	6.57 × 10^–3^	0.092	1.89 × 10^–1^	0.127	3.05 × 10^–3^
CB fore	0.143	3.51 × 10^–3^	0.049	4.35 × 10^–1^	0.108	5.16 × 10^–3^

*CB*, coiled bodies; *TA*, tufted astrocyte; *NFT*, neurofibrillary tangle; fore, forebrain structures; hind, hindbrain structures

**Fig. 4:**
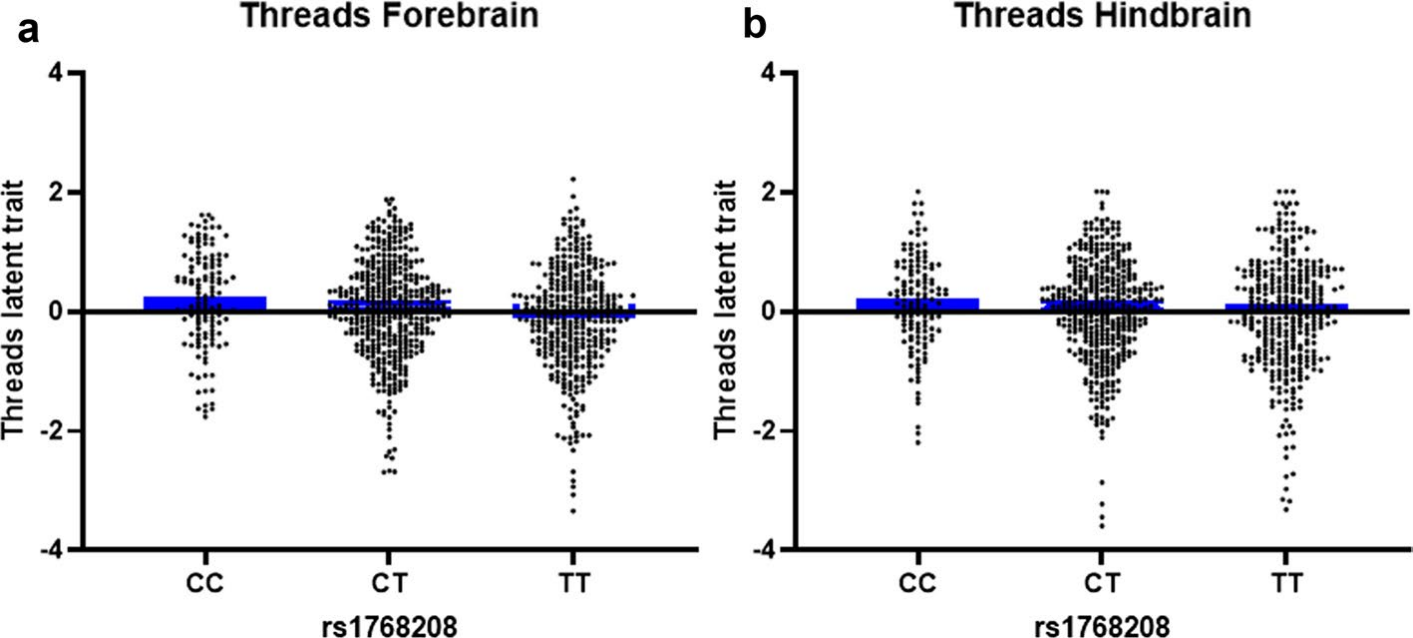
Latent trait scores stratified by rs1768208 genotypes. LT measures were generated from semi-quantitative scores of tau-immunoreactive threads in a large PSP cohort and plotted stratified by rs1768208. Each data point represents the LT score for a PSP case. These LT measures were used as phenotypes in linear regression analysis. The top PSP GWAS SNP, rs17682208, is associated with tau thread pathology variability The “C” risk allele is associated with a decrease in tau thread burden compared to the “T” major allele in the forebrain (**a**) and hindbrain (**b**) in PSP. LT, latent trait; Blue bars represent the median

Testing for association with PSP LTs at the *MOBP* locus (rs1768208) identified a significant LT/SNP association with rs1768208 and forebrain threads (*P* = 2.14 × 10^–5^, Beta = 0.169) (Fig. 4a), overall threads (*P* = 4.65 × 10^–4^, Beta = 0.145), and forebrain CBs (*P* = 5.16 × 10^–3^, Beta = 0.108) (Table 2). These top associations show that rs1768208/LT is associated with increased tau thread load in hindbrain and forebrain with each copy of the “C” minor allele (Fig. 4a, b). The remaining PSP susceptibility loci at *MAPT* (H1H2 haplotype-tagging SNP), *STX6*, *EIF2AK3*, *SLCO1A2*, and *DUSP10* showed suggestive associations with LTs in PSP (Online Resource Table 2).

### Genome-wide association analysis with PSP tau and MOBP burden

Regions of interest were selected from the overall LT information plot based on the area under the curve per brain region (Fig. 2b). The midbrain tectum and red nucleus were sampled at the level of the oculomotor nerve (Online Resource Fig. 2). Slides with CP13 immunohistochemistry were scanned to high-resolution digital images, midbrain tectum and red nucleus were annotated, and annotation were subjected to a custom color deconvolution macro to quantify a percent tau burden. A subset of PSP cases were selected from the initial cohort based on the availability of CP13 slides. Linear regression was employed to test for association between genome-wide genotyping using age and sex as covariates. SNPs that overlapped with PSP LT results were further tested for association in the stage 2 cohort of PSP cases with CP13 slides available (*N* = 375), and meta-analysis was performed (Table 3). Interestingly, the top SNP common across LT and tau QT associations was rs1768208 at *MOBP*. Full summary statistics for stage 1 PSP QT GWAS of midbrain tectum and red nucleus for SNPs *P* < 10^–3^ are available in Online Resource Tables 3 and4.

**Table 3:** Digitally quantified tau pathology burden genetic associations in PSP

		Midbrain tectum						Red nucleus					
		Stage 1		Stage 2		Meta-analysis		Stage 1		Stage 2		Meta-analysis	
CHR	SNP	BETA	*P*	BETA	*P*	Beta	*P*	BETA	*P*	BETA	*P*	BETA	*P*
3	rs1768208	0.211	5.02E−04	0.105	2.70 E−02	0.151	4.03 E−05	0.211	5.02 E−04	0.171	3.13 E−03	0.190	4.78 E−06
2	rs6723687	− 0.666	1.26 E−05	0.144	3.81 E−01	− 0.402	2.25 E−04	− 0.666	1.26 E−05	0.254	2.04 E−01	− 0.332	5.70 E−03
3	rs7433256	− 1.577	3.67 E−06	− 0.035	8.52 E−01	− 0.585	2.99 E−04	− 1.577	3.67 E−06	− 0.015	9.49 E−01	− 0.512	6.91 E−03
8	rs11203544	− 0.433	6.03 E−04	-0.076	3.62 E−01	− 0.211	2.18 E−03	− 0.433	6.03 E−04	− 0.118	2.49 E−01	− 0.243	2.09 E−03
4	rs2048542	− 0.336	8.27 E−06	− 0.047	4.38 E−01	− 0.142	2.43 E−03	− 0.336	8.27 E−06	0.029	6.97E− 01	− 0.153	3.56 E−03
6	rs12203224	0.214	3.75 E−04	0.010	8.38 E−01	0.105	5.39 E−03	0.214	3.75 E−04	0.063	2.98 E−01	0.140	1.00 E−03
4	rs17470661	− 0.401	4.64 E−06	− 0.038	6.01 E−01	− 0.145	8.71 E−03	− 0.401	4.64 E−06	0.020	8.18 E−01	− 0.195	1.58 E−03
6	rs9395145	− 0.501	3.96 E−04	0.106	4.00 E−01	− 0.182	4.99 E−02	− 0.501	3.96 E−04	0.198	1.97 E−01	− 0.183	7.76 E−02
2	rs6543017	− 0.392	8.43 E−06	0.039	5.77 E−01	− 0.103	6.05 E−02	-0.392	8.43 E−06	− 0.081	3.48 E−01	− 0.236	1.20 E−04
7	rs10486848	− 0.328	1.18 E−05	0.108	5.97 E−02	− 0.074	9.74 E−02	-0.328	1.18 E−05	0.124	7.69 E−02	− 0.089	7.97 E−02
6	rs2022421	− 0.535	1.98 E−04	0.200	1.11 E−01	− 0.153	1.01 E−01	− 0.535	1.98 E−04	0.238	1.21 E−01	− 0.176	9.11 E−02
4	rs1512315	− 0.426	3.92 E−06	0.032	6.47 E−01	− 0.089	1.06 E−01	− 0.426	3.92 E−06	0.075	3.80 E− E−01	− 0.158	1.08 E−02
7	rs2732744	− 0.262	1.69 E−04	0.094	9.75 E−02	− 0.046	2.93 E−01	− 0.262	1.69 E−04	0.103	1.38 E−01	− 0.080	1.01 E−01
7	rs1029563	− 0.338	2.01 E−06	0.149	5.40 E−03	− 0.042	3.22 E−01	− 0.338	2.01 E−06	0.161	1.34 E−02	− 0.069	1.46 E−01
7	rs2732757	− 0.259	1.99 E−04	0.116	3.91 E−02	− 0.029	4.97 E−01	− 0.259	1.99 E−04	0.121	7.83 E−02	− 0.068	1.64 E−01

*Chr*, chromosome; *SNP*, single nucleotide polymorphism

**Fig. 5:**
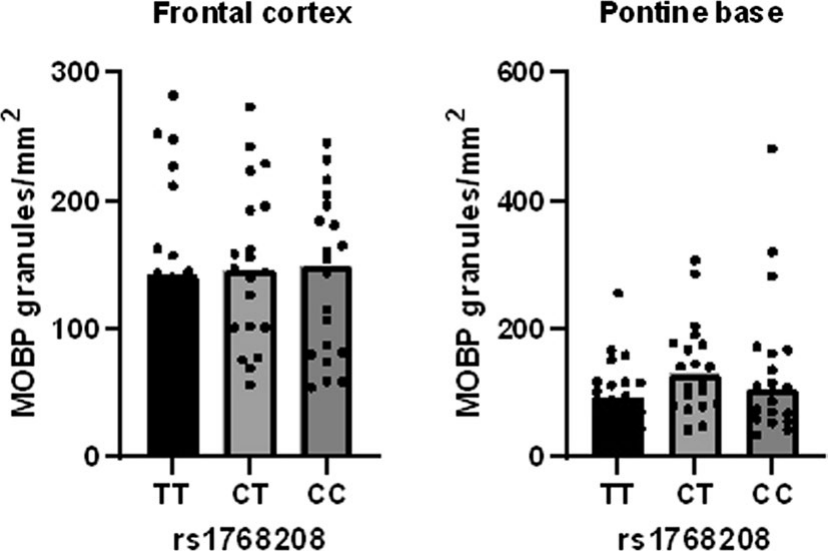
Quantification of MOBP granular inclusion in PSP. Tissue sections from superior frontal cortex and pons were subjected to MOBP immunohistochemistry, digitally scanned, and analyzed using a custom-designed macro to detect and quantify MOBP granular inclusions. Frontal cortex (left) and pons (right) are stratified by the top PSP GWAS SNP rs1768208 genotype. MOBP, myelin-associated oligodendrocytic basic protein

Since *MAPT* and *MOBP* genotypes are associated with tau pathology heterogeneity, we next sought to determine whether there is a genotype effect on MOBP pathology. MOBP is a component of the compact myelin sheath, is highly expressed in CNS white matter, and forms small granules in white matter of PSP (Online Resource Fig. 3). We selected superior frontal cortex to represent forebrain and pons to represent hindbrain. Digital image analysis to quantify MOBP granules in frontal cortex and pontine base did not detect a significant difference in MOBP granules across the various rs1768208 genotypes (Fig. 5).

### Genome-wide association analysis with PSP latent traits

The initial PSP cohort (*N* = 481) with genome-wide genotyping and tau pathology LTs, were tested for LT/SNP associations using linear regression under an additive model and age and sex as covariates. This identified 62 SNPs associated with PSP LTs (*P* < 10^–5^). Each quantitative trait locus identified was further scrutinized for LD structure, and of the 62 SNPs, 37 were selected to represent these top loci for further genotyping in an extended PSP cohort (*N* = 401). These analyses were also analyzed by linear regression using an additive model and age and sex as covariates, and then a combined analysis was performed in PLINK. Top SNP/LT associations (Table 4) were identified at rs3088159 (located in intron 4 of *RP11-23P13.6*, a non-coding RNA between *SPTBN5*/*EHD4*) with overall NFTs (*P* = 1.26 × 10^–6^, Beta = − 0.221), rs2305196 (*HK1*) with hindbrain threads (*P* = 2.72 × 10^–6^, Beta = − 0.198), rs154239 (*SEC13*) with overall threads, and rs6543017 (*TBC1D8*) with forebrain CBs (*P* = 6.66 × 10^–6^, Beta = − 0.252). These SNP/LT associations do not pass the Bonferroni correction for multiple testing. The complete results are presented in Online Resource Table 5. The most significant SNP/LT associations were identified at genetic loci that have not been previously implicated in PSP, but could potentially be biologically-related to genes known to be involved in PSP pathogenesis based on PANTHER protein family/function analysis (Online Resource Table 6) [36].

**Table 4:** PSP latent trait GWAS results

rsID	Chr	Latent trait	Position^a^	Alleles	MAF^b^	MAF^c^	Beta	*P* value^d^	Candidate gene(s)
rs3088159	15	NFT	42190692	G > A	0.27	0.29	− 0.22	1.26 E−06	*SPTBN5*/*EHD4*
rs154239	3	Threads	10364793	C > A	0.37	0.35	− 0.20	2.78 E−06	*SEC13/ATP2B2*
rs6762033	3	Overall	135337113	T > C	0.14	0.14	− 0.27	1.20 E−05	*EPHB1/PPP2R3A*
rs6543017	2	CB	101698782	A > G	0.12	0.12	− 0.27	1.36 E−05	*TBC1D8*
rs1333619	6	TA	137732135	G > A	0.19	0.16	− 0.23	1.46 E−05	*IFNGR1/OLIG3*
rs6771438	3	Overall	186682324	T > G	0.11	0.11	− 0.30	1.53 E−05	*ST6GAL1*
rs2305196	10	Threads	71144324	C > T	0.28	0.25	− 0.19	2.19 E−05	*HK1*
rs16904149	8	NFT	91229254	T > G	0.06	0.05	− 0.33	7.54 E−05	*CALB1/LINC00534*
rs11203544	8	Overall	13741215	C > T	0.07	0.08	− 0.34	8.22 E−05	*LOC102725080/SGCZ*
rs17470661	4	Threads	45083659	G > A	0.13	0.15	− 0.23	9.13 E−05	*RP11-362I1.1*

*Chr*, chromosome; *MAF*, minor allele frequency; *NFT*, neurofibrillary tangles; *CB*, coiled bodies

^a^Position based on GRCh37.p13 assembly

^b^Minor allele frequency in PSP combined cohort

^c^Minor allele frequency obtained from 1000 Genomes Project Phase 3 (32) European population (www.1000genomes.org/)

^d^Combined analysis *p* value

Subsequently, we performed genetic imputation against HRC panel for variants with MAF > 2% and R^2^ ≥ 0.90, which resulted in ~ 6 million variants. Imputed genotypes were tested for genetic association with PSP LTs by linear regression under an additive model using age at death, sex, and the first four principle components for population substructure as covariates. This identified SNP/LT associations largely overlapping with previous results with the exception of one, rs2294892, which was significantly associated with hindbrain tau burden (*P* < 9 × 10^–8^). The SNP rs2294892 was then genotyped in 400 PSP cases comprising stage 2 cohort and meta-analysis was performed. The imputed SNP rs2294892 was nominally associated with hindbrain tau (*P* = 1.31 × 10^–5^).

### Brain transcriptome analysis

We next sought to determine whether the 10 top PSP LT GWAS SNPs regulate expression of nearby genes. SNP/transcript associations were tested on 349 Alzheimer’s disease, PSP, and pathological aging temporal cortex and cerebellum RNA samples as previously described [2, 4]. SNPs within ± 1 Mb of the Ensembl gene locus were tested for association with CQN gene expression levels using a linear mixed model implemented with the R *lme4* package. This identified significant SNP/transcript associations, or eQTLs, at rs3088159 and rs154239 (Table 5). The SNP rs3088159 allele dosage significantly associated with *SPTBN5* in temporal cortex (*Q* = 3.22 × 10^–4^, Beta = − 0.212) and cerebellum (*Q* = 5.41 × 10^–3^, Beta = 0.237), and *EHD4* in cerebellum (*Q* = 2.64 × 10^–5^, Beta = 0.231). The SNP that was identified to associate with tau threads, rs154239, showed significant associations with *GHRL* in temporal cortex (*Q* = 1.91 × 10^–3^, Beta = − 0.203) and cerebellum (*Q* = 3.99 × 10^–5^, Beta = − 0.283).

**Table 5:** Brain transcriptome association with PSP LT GWAS SNPs

	Gene	GeneID	SNP	A1	A2	BETA	SE	*P*	*Q*
Temporal cortex	*SPTBN5*	ENSG00000137877	rs3088159	T	C	− 0.212	0.042	8.51 E−07	3.22 E−04
	*GHRL*	ENSG00000157017	rs154239	T	G	− 0.203	0.044	6.40 E−06	1.91 E−03
	*RPAP1*	ENSG00000103932	rs3088159	T	C	0.040	0.015	7.97 E−03	0.38
	*PCCB*	ENSG00000114054	rs6762033	C	T	− 0.069	0.030	2.49 E−02	0.58
	*NECAB1*	ENSG00000123119	rs16904149	G	T	− 0.162	0.072	2.66 E−02	0.59
	*VPS39*	ENSG00000166887	rs3088159	T	C	− 0.033	0.015	3.15 E−02	0.62
Cerebellum	*EHD4*	ENSG00000103966	rs3088159	T	C	0.231	0.041	5.97 E−08	2.64 E−05
	*GHRL*	ENSG00000157017	rs154239	T	G	− 0.283	0.051	9.45 E−08	3.99 E−05
	*SPTBN5*	ENSG00000137877	rs3088159	T	C	0.237	0.055	2.51 E−05	5.41 E−03
	*RPAP1*	ENSG00000103932	rs3088159	T	C	0.045	0.015	3.69 E−03	0.23
	*ST6GAL1*	ENSG00000073849	rs6771438	G	T	− 0.206	0.081	1.18 E−02	0.41
	*MAPKBP1*	ENSG00000137802	rs3088159	T	C	0.074	0.031	1.83 E−02	0.49
	*RP11*-*211G3.3*	ENSG00000228804	rs6771438	G	T	− 0.166	0.072	2.20 E−02	0.53
	*PPA1*	ENSG00000180817	rs2305196	A	G	0.060	0.026	2.45 E−02	0.55
	*CCAR1*	ENSG00000060339	rs2305196	A	G	− 0.048	0.022	3.62 E−02	0.61
	*IL17RC*	ENSG00000163702	rs154239	T	G	− 0.072	0.034	3.81 E−02	0.62
	*RUFY2*	ENSG00000204130	rs2305196	A	G	− 0.048	0.023	4.36 E−02	0.65
	*SEC13*	ENSG00000157020	rs154239	T	G	0.035	0.017	4.56 E−02	0.65

SNP, single nucleotide polymorphism; A1, allele 1; A2, allele 2; SE, standard error; Q, FDR adjusted *P* value

## Discussion

Here we have taken an innovative approach to summarize tau neuropathology in PSP by applying a statistical model principally used in psychometric testing. With the enormous amount of data for 900 cases, 18 brain regions, and 4 brain lesions, this LT modeling was a way to reduce the complex dataset into single, representative values to use as quantitative traits in a GWAS. In doing so, we were able to assign an intermediate phenotype score to each individual PSP case to test for association with their genetic makeup. These LT/SNP associations did not meet the conservative Bonferroni genome-wide significant threshold, but this is most likely due to the small sample size. One may expect to see a strong association with the tau H1H2 haplotype-tagging SNP, rs8072553, however the H2 allele frequency is too low in PSP to discern a difference in LT/neuropathologic variability.

The LT association analysis results with PSP GWAS top susceptibility loci provide important insight into the methodology employed in this study. For example, the “A” risk allele is associated with lower hindbrain CB burden, but a higher TA hindbrain burden (Fig. 3a,b). Regionally, the positive beta values show that the “A” risk allele is associated with higher loads of TA lesions in the hindbrain, whereas the rs242557 association with forebrain TAs had negative beta values, suggesting that rs242557 imposes brain region-specific changes in *MAPT* expression in PSP. The *MAPT* locus has been extensively studied [37, 40, 43], and furthermore, there have been large SNP/transcript level associations studies performed for the top PSP GWAS SNPs in human brain tissue [20, 52]. As we recently reported in a smaller PSP cohort, the most significant associations with rs242557 and PSP LTs were with glial lesions [3]. The rs242557 H1c *MAPT* haplotype-tagging SNP is located in a highly conserved regulatory region in *MAPT* intron 0. There have been conflicting results using luciferase reporter assays showing that both the “A” risk allele and “G” protective allele can cause an increase luciferase activity [37, 43]. Regardless of these discrepancies, rs242557 does contribute to the regulation of *MAPT* expression levels, and the LT/rs242557 association shows different effects on lesion type burden comparing CBs to TAs, and in a regional-dependent manner for TAs (Table 2, Fig. 3).

Importantly, these results are in agreement with our current understanding of the H1c sub-haplotype association with the risk of developing PSP. With the identification of rs242557/PSP risk, Rademakers et al. stratified PSP cases by age, and found that PSP patients with an age at death ≤ 75 years had an increased risk allele frequency (0.62) compared to PSP patients ≥ 76 at age of death (0.56)[43]. Furthermore, hindbrain-predominant PSP patients are those more likely to die at a younger age compared to the forebrain-predominant patients, due to the greater affection of key brainstem nuclei required for sustaining life relative to higher cortical functions. With our finding that the “A” risk allele is associated with a higher TA burden in the hindbrain, and the increased frequency of the “A” allele in younger PSP cases, we sought to further understand the relationship between these results. Upon stratifying our PSP cohort into hindbrain- or forebrain-predominant based on available clinical presentations (70% of the combined cohort, *N* = 622), consistent differences were identified based on the present knowledge of the H1c sub-haplotype risk and genetic/neuropathologic/clinical features in PSP. The hindbrain PSP patients (*N* = 77) were younger at age of death (mean 73.3 years) compared the forebrain PSP patients (*N* = 96) (mean 77.2 years), and yet typical PSP patients (*N* = 449) had a mean age at death of 73.7 years.

Another PSP risk locus at *MOBP* (rs1768208) encodes myelin oligodendrocytic basic protein, a CNS-specific component of myelin, was associated with tau pathology LTs, and more specifically to glial lesion LTs. Based on SNP/transcript studies in human brain tissue, the “T” risk allele for rs1768208 is associated with increased *MOBP* expression levels [20, 52]. Comparable to our interpretation of rs242557/LT results, the rs1768208/LT associations show that with each copy of the minor allele, there is an increase in tau threads and CB load (*i.e*. positive beta value).

The novel loci we identified by GWAS may play an important role in PSP based on our current knowledge of SNP/transcript associations for rs242557 and rs1768208 and the neuropathologic associations identified in the present study. The fact that we were able to detect significant SNP/transcript associations with rs3088159 and rs154239 suggest these have a functional role for the genetic associations we identified. Interestingly, Gene Ontology analysis shows that 46% (6/13) of the gene products identified to associate with tau pathology LTs are localized to extracellular exosomes (GO:0070062), which include CALB1, EHD4, EPHB1, ST6GAL1, SEC13, and SPTBN5. The SNP rs1411478 at syntaxin 6 (*STX6*) was identified in the PSP GWAS as a risk locus, and is involved in vesicle-mediated transport in the endocytic and exocytic pathways [8, 50]. In order to maintain cellular homeostasis, there is a critical interplay of cytoskeletal components including microtubule and actin filaments with motor proteins and membrane-associated vesicle proteins. The loci we identified here in the PSP LT GWAS are enriched in these types of membrane-cytoskeletal proteins along with evidence to support the existence of direct interactions. For example, spectrin beta chain, non-erythrocytic 5 (SPTBN5) and tau are both cytoskeletal proteins and it has been reported that tau inhibits F-actin crosslinking activity of spectrin [9]. More recently, Krieg et al*.* showed that the combination of spectrin and microtubules provide axonal and dendritic protection from mechanical stress in *C. elegans* with mutations in β-spectrin and tau homologues [28]. Additionally, *SPTBN2* mutations have been identified to cause spinocerebellar ataxia type 5 via a mechanism involving glutamate receptors, setting precedence for mutant spectrin protein to cause neurodegeneration [21]. The SNP/transcript association at rs3088159 was also associated with *EHD4* transcript levels (Table 5). Eps15 Homology Domain Protein (EHD) family protein 4, or EHD4, was originally identified as a extracellular matrix protein [29]and found later to play an important role in endosomal recycling and is involved in the control of trafficking at the early endosome, regulating exit of cargo toward both the recycling compartment and the late endocytic pathway [47]. Taken together, our results suggest that extracellular exosomes play an important role in PSP disease pathogenesis.

Some of the novel SNP/LT loci are known to be involved in Alzheimer’s disease (AD), neuronal health, and tau phosphorylation. Calbindin (CALB1) is a calcium-binding protein reported to be involved in AD, and plays an important role in preventing neuronal death [17, 23]. Protein phosphatase 2 regulatory subunit B''alpha (PPP2R3A) is one of the subunits of protein phosphatase 2, which as a major Ser/Thr phosphatase, has been extensively studied in the context of AD and tau phosphorylation homeostasis [22]. The tyrosine kinase ephrin B1 (EPHB1) is also interesting candidate gene that may be involved in PSP pathogenesis. The interplay between EPHB1, EPHB2, and GSK-3β plays a significant role in regulating tau phosphorylation levels [24].

In conclusion, using tau neuropathologic heterogeneity as intermediate phenotypes in PSP is a novel approach to identifying QTLs. Testing for genetic association with a biological measurement within a disease cohort, rather than a case–control genetic association study, may facilitate the identification of biological mechanisms underlying disease pathogenesis.

## Supplementary Information

Below is the link to the electronic supplementary material.

Supplementary file1 (XLSX 196 KB)

Supplementary file2 (XML 12 KB)

Supplementary file3 (PDF 2930 KB)
