# Supplementary material for: Latent trait modeling of tau neuropathology in progressive supranuclear palsy
Source: Acta Neuropathol. 2021 Feb 26;141(5):667–80. doi: 10.1007/s00401-021-02289-0 (PMC8043857; doi:10.1007/s00401-021-02289-0)
Supplement: Supplementary file 3 — Supplementary file3 (PDF 2930 KB) [file 401_2021_2289_MOESM3_ESM.pdf]

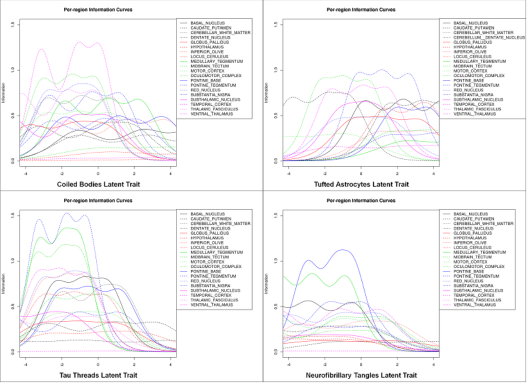

Online Resource Figure 1. Item Information Curves for PSP per tau lesion. Item Information Curves are generated with the R ltm package. These provide the contribution of each brain region per tau lesion captured by the latent trait variables.

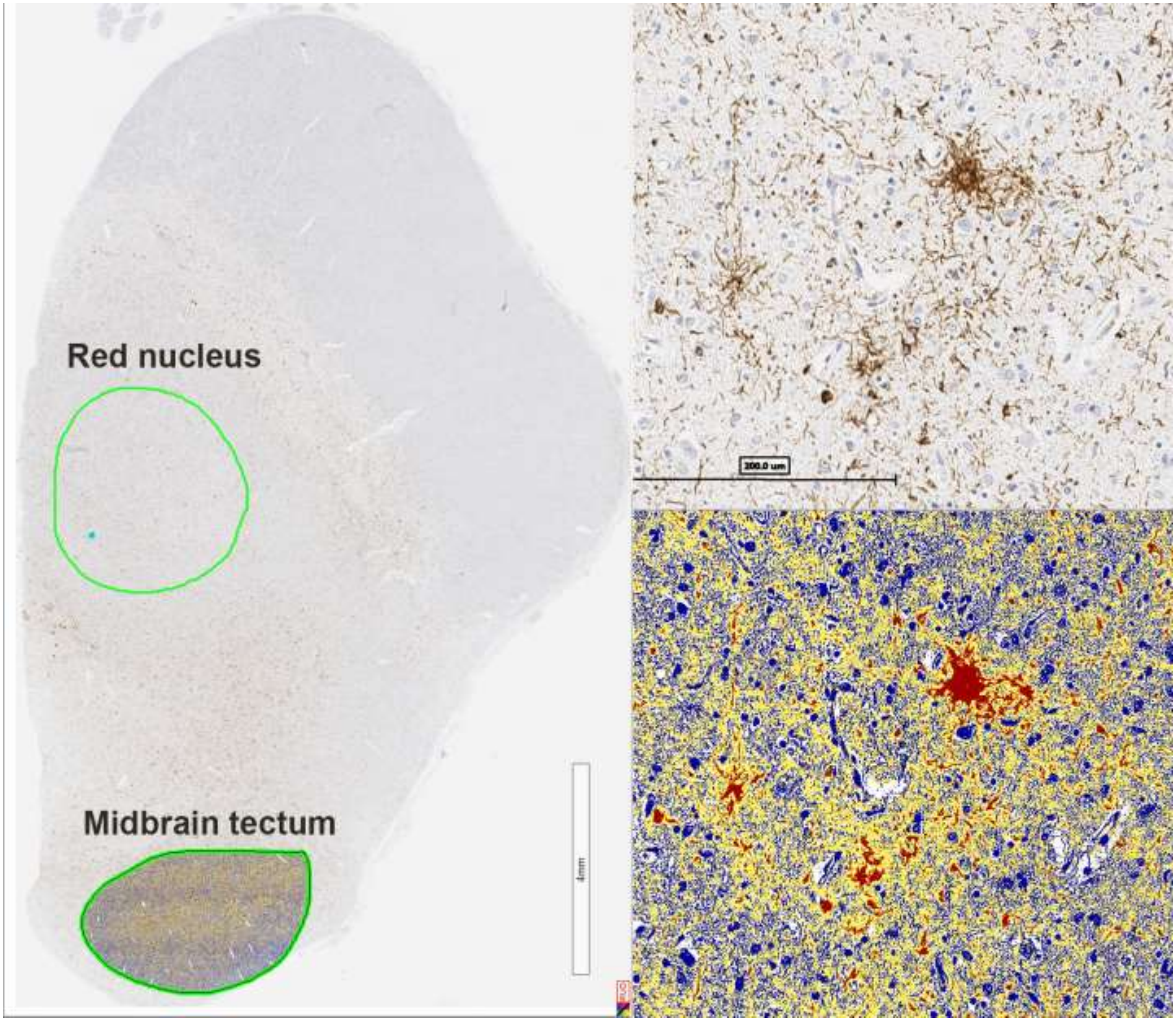

Online Resource Figure 2. Digital microscopy and image analysis in PSP. For quantitative analysis of phosphorylated tau burden, sections from the midbrain at the level of the 3rd cranial nerve were immunostained with CP13 (Dr. Peter Davies). Slides are scanned with an Aperio ScanScope AT2 and converted to high-resolution digital images. A custom color deconvolution (v9) was adapted using ImageScope to quantify DAB-positive chromogen representing phosphorylated and generates an overall percent burden to be used in genetic association studies.

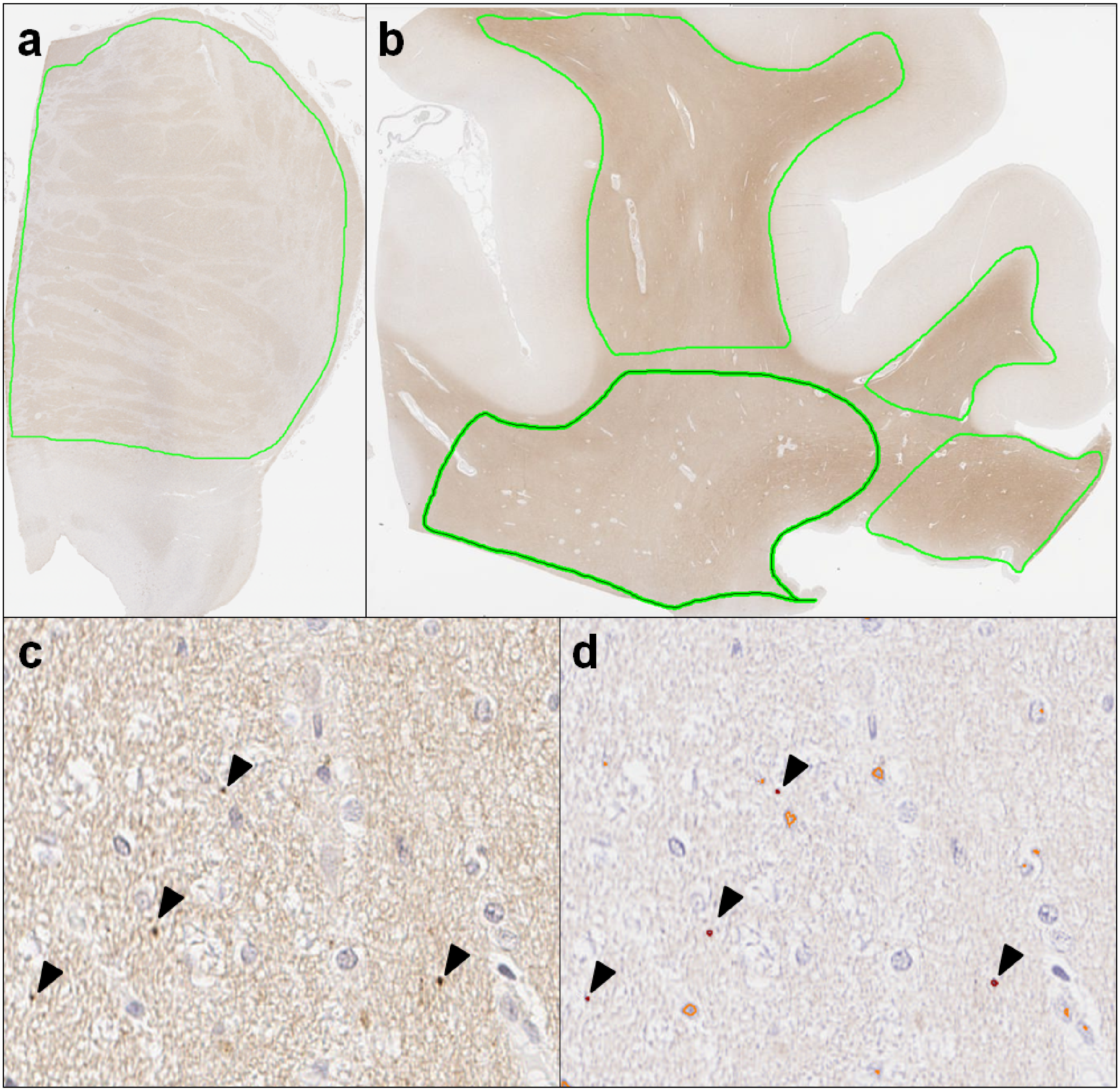

Online resource Fig 3. Myelin oligodendrocytic basic protein (MOBP) Immunohistochemical analysis in PSP. Sections from pons (a) representing the hindbrain and superior frontal cortex (b) representing the forebrain were analyzed by digital image analysis in order to determine whether there is an association between rs1768208 genotype and MOBP granule burden. Regions of interest include pontine base (a) and the mean of four regions was calculated for the superior frontal cortex and included the corpus callosum, cingulate white matter, superior frontal white matter, and deep frontal white matter (b). Inset from pontine base (c) shows small, MOBP-immunoreactive granules in white matter (arrowheads) and the IHC nuclear macro markup image post-analysis identifies granules as 3+ (red) positive “nuclei” quantifying the granules per mm^2.
